# Combined application of up to ten pesticides decreases key soil processes

**DOI:** 10.1007/s11356-024-31836-x

**Published:** 2024-01-16

**Authors:** Peter Meidl, Anika Lehmann, Mohan Bi, Carla Breitenreiter, Jasmina Benkrama, Erqin Li, Judith Riedo, Matthias C. Rillig

**Affiliations:** 1 Institut Für Biologie, Freie Universität Berlin, Berlin, Germany; 2 Berlin-Brandenburg Institute of Advanced Biodiversity Research (BBIB), Berlin, Germany

**Keywords:** Agriculture, Currently used pesticides, Litter decomposition, Rate of change, Soil processes, Water stable aggregates

## Abstract

Natural systems are under increasing pressure by a range of anthropogenic global change factors. Pesticides represent a nearly ubiquitously occurring global change factor and have the potential to affect soil functions. Currently the use of synthetic pesticides is at an all-time high with over 400 active ingredients being utilized in the EU alone, with dozens of these pesticides occurring concurrently in soil. However, we presently do not understand the impacts of the potential interaction of multiple pesticides when applied simultaneously. Using soil collected from a local grassland, we utilize soil microcosms to examine the role of both rate of change and number of a selection of ten currently used pesticides on soil processes, including litter decomposition, water stable aggregates, aggregate size, soil pH, and EC. Additionally, we used null models to enrich our analyses to examine potential patterns caused by interactions between pesticide treatments. We find that both gradual and abrupt pesticide application have negative consequences for soil processes. Notably, pesticide number plays a significant role in affecting soil health. Null models also reveal potential synergistic behavior between pesticides which can further their consequences on soil processes. Our research highlights the complex impacts of pesticides, and the need for environmental policy to address the threats posed by pesticides.

**Supplementary Information:**

The online version contains supplementary material available at 10.1007/s11356-024-31836-x.

## Introduction

### Soil, global change factors, and pesticides

Soils harbor key biogeochemical processes that drive the functioning of terrestrial ecosystems, including organic matter decomposition, cycling of nutrients, and soil aggregation (Bardgett and van der Putten 2014; Wagg et al. 2014; Schimel 2016). Recently, the International Panel on Climate Change has reported the extreme duress faced by global ecosystems due to the effect of multiple environmental change factors, and the ever-increasing vulnerability of these systems as the impacts of these factors accumulate (IPCC 2022). Although organisms in nearly any given system are likely to have experienced a historical disturbance regime, in which a system is subjected to a stress, followed by a period of recovery, the pressure of added elements of global change can threaten to break the resilience of some species, pushing them past a recoverable threshold, and thus potentially altering ecosystem processes permanently. With the consequences of global change factors reaching an increasing number of ecosystems, it is likely that many fundamental soil processes including decomposition, nutrient cycling, and aggregation are placed under sustained pressure, placing already vulnerable systems under more strain.

One global change factor that has become nearly ubiquitous in its occurrence in agriculture and beyond is pesticides. These chemicals are regularly applied on agricultural fields to repress the impact of crop pathogens or pests. Although the application of pesticides has been practiced for centuries, the ecological consequences they often cause have not waned with the advent of new pesticides, and the global use of synthetic pesticides has increased by 80% between 1990 and 2017 (Kenea 2021; Zhang et al. 2011). In the European Union (EU) alone, there are over 400 active ingredients registered to be applied in currently used pesticides, with many agricultural systems dependent on their regular application for commercially viable harvests (European Commission 2020). Between 2011 and 2018, EU rates of pesticide sales have remained at around 360,000 kt (Eurostat 2020). The use of many of these pesticides significantly impacts the functioning of air, water, and soil systems, and ultimately these systems’ ability to maintain integral ecosystem services including nutrient cycling and aggregate formation (Lautenbach et al. 2012; Silva et al. 2019). Furthermore, pesticides have been shown to drastically exacerbate biodiversity loss in both aquatic and terrestrial systems, including ecologically key species such as pollinators and earthworms (Isenring 2010; Pelosi et al. 2014). Finding multiple pesticides in soil is a common phenomenon: in agricultural topsoils throughout the EU, more than 51% of soils contained more than five pesticides (Silva et al. 2019); in managed grassland soils, over 58% of soils contained two or more pesticide residues (Hvězdová et al. 2018); and in organically managed arable and vegetable fields, 85% of soils contained five or more pesticide residues (Riedo et al. 2022). Soil biota such as earthworms and nematodes in both agricultural and non-agricultural sites have also been shown to contain a variety of pesticides, pointing towards extensive environmental contamination (Pelosi et al. 2021). Therefore, despite being lauded for their biodegradability, pesticides form chronically toxic residues in soil that can exist in both soil and target crops for years after initial exposure (Bošković et al. 2020; Kaur et al. 2017). Yet, despite reports of numerous pesticides occurring in soils and soil biota, we know little about potential impacts of multiple pesticides on soil processes.

### Rate of change

In addition, we also do not understand the effects of the rate of appearance of pesticides in soils. The seasonal or annual application and subsequent spread of these chemicals via several processes including drift of pesticides during application, runoff, and atmospheric volatilization ultimately cause off-target displacement which occurs gradually over time (Galon et al. 2021; Tang et al. 2021; Riedo et al. 2022). Furthermore, factors of global change such as increased temperature have caused some areas to experience increases in pest populations, which has driven use of various pesticides, resulting in increasing threats to surrounding systems (Hatfield et al. 2011). Despite this, experiments typically only study the effects of (abrupt) additions of chemicals. Existing studies regarding rate of change dynamics have revealed that both organisms and whole systems respond differently to gradual vs. abrupt environmental change (Golubeva et al. 2001; Li et al. 2021; Pinek et al. 2020). Key components to consider when applying rate of change treatments are the ramping time and magnitude of treatments (Pinek et al 2020). For instance, an abrupt treatment would receive the dosage of a given treatment all at once, while a gradual treatment would experience the same dose over a longer ramp time prior to reaching the treatment’s peak magnitude. Because the impacts of pesticides can occur at different rates due to application and gradual buildup of pesticides due to displacement, it is vital to investigate both the number and rate of addition of pesticides to understand effects on soil processes.

### Hypotheses

Here we report on a study examining the impacts of pesticide number and application rate on critical soil processes. We tested ten single pesticides individually, as well as mixtures of five and ten pesticides. We applied these pesticides both abruptly and gradually to soil mesocosms. We hypothesized that the impact of single pesticides would vary distinctly due to their specific mode of action, but that the combined effects of five and ten pesticides will deviate from mere addition of effects of the single pesticides. Additionally, we expect that abrupt application of pesticides will more drastically affect soil processes in comparison to gradual application, due to microbes having less time to adjust to pesticide-induced stress. We provide evidence regarding the effects of multiple pesticides and their application rate on litter decomposition rate and soil aggregation, as well as on soil properties, such as soil pH and electrical conductivity (EC).

## Methods

### Pesticide selection

To assess the impacts of multiple pesticides and rate of application on soil processes, we designed a microcosm experiment using a pool of ten commonly occurring pesticides. We used ten pesticides that included two insecticides, four fungicides, and four herbicides. These were selected based on the analysis of 280 conventionally managed agricultural fields for pesticide occurrence and persistence in fields using data from an EU-wide soil survey (Pelosi et al. 2021; Riedo et al 2021). Pesticide concentrations were selected based on their maximal measured concentrations across all 280 study sites (Table 1).

**Table 1:** The ten pesticides used in this study. Concentrations of 10 pesticides in 280 soils, ordered by decreasing numbers of occurrence and maximal concentration (Pelosi et al. 2021; Riedo et al. 2021)

Name	Type	Maximal Concentration (ng a.i. g^−1^soil)	Percentage of detection (%)
Imidacloprid (Imi)*	Insecticide	150	78%
Epoxiconazole (Epo)*	Fungicide	300	73%
Boscalid (Bos)	Fungicide	1200	64%
Diflufenican (Dif)	Herbicide	1300	58%
Napropamide (Nap)	Herbicide	100	57%
Cyproconazole (Cyp)	Fungicide	250	40%
S-metolachlor (Sme)	Herbicide	80	39%
Metrafenone (Met)	Fungicide	200	22%
Pendimethalin (Pen)	Herbicide	1000	20%
Clothianidin (Clo)*	Insecticide	60	20%

*Not currently approved in EU (emergency authorization)

We used the EU pesticides database (EU 2023) to check current approval status. Substances marked with asterisks are not currently approved, but all of them are under emergency authorizations in some European countries, which means they can all be used at present.

### Soil sampling and pre-treatment

Soil was collected from a local grassland at an experimental site of Freie Universität Berlin (52°46′60.67 N, 13°30′26.98 E) using the top 10 cm of surface soil (the litter layer was removed prior to sampling). Soil was then sieved to 2 mm and subsequently stored at 22 ℃ for 5 h to reduce soil moisture to below 60% water-holding capacity. Soil was then weighed into 30 g increments and filled into microboxes (Deinze, Belgium; filter lid, 80 mm diameter, 40 mm height). Before starting the experiment, soil moisture in the microboxes was adjusted to 60% water-holding capacity by addition of deionized water. To investigate the consequences of pesticide treatments on litter decomposition, we created miniature tea bags (Keuskamp et al. 2013). A 30-µm nylon mesh (Sefar Nitex) was cut into 2.5 cm × 3 cm rectangles, folded, and both 1.5 cm long sides closed via an impulse sealer (Mercier Corporation, product no. 127174). The bags were filled with 300 mg green tea (Lipton green tea, Sencha Exclusive Selection), sealed, autoclaved (121 °C for 20 min in a dry cycle), and dried at (60 °C) before they were introduced to the test system.

### Pesticide preparation

To assess potential effects of solvents, we tested the final targeted concentration of acetone (0.1%, v/v), tween (0.16%), water alone, and a combination of all three solvents as individual treatments. Primary stock solutions were prepared in 99% acetone. These solutions were then stored at 4 ℃. Individual working solutions per pesticide were created for a 1000 × dilution for the abrupt treatments, and gradual treatments were diluted using this concentration but subsequently diluted to ${~}^{1}\!\left/ \!{~}_{5}\right.$ of the abrupt treatment solutions, to allow for identical pesticide application between the abrupt and gradual treatments. Dilutions for working solutions were made in 0.1% acetone solution.

### Pesticide application

Pesticide treatments were applied: individually (abruptly *n* = 10, gradually *n* = 10), as mixtures of five (abruptly *n* = 20, gradually *n* = 20), or all ten pesticides simultaneously (abruptly *n* = 20, gradually *n* = 20) with the same dose applied regardless of treatment. For the five pesticide treatments, pesticides were chosen randomly, and thus each replicate received a different combination of five pesticides (Table S3). A 1-mL glass syringe with a nasal spray pump (LMA MAD 300 needle-free drug delivery device; Teleflex Medical Europe Ltd., Athlone Co. Westmeath, Ireland) was used to apply pesticide treatments directly to the soil’s surface to avoid loss. Abrupt and gradual pesticide application received the same concentration of pesticides over the same period, but the starting concentration differed for each since a stepwise buildup of contamination was necessary for the gradual treatment. The principle of application is to maintain the same area under the curve for both treatments (Figure S1). Therefore, gradual treatments were applied every 10 days for the 50-day experimental duration, and the abrupt treatment was applied in a single dose on day 20. For the gradual treatment, a 0.5 mL mixture of working solution and 0.5 mL diluted water (overall 1 mL) was added every 10 days. Then, we added 0.5 mL of acetone with targeted concentration (0.02%, v/v) and 0.5 mL diluted water (overall 1 mL) into control every 10 days and 1 mL of diluted water into abrupt treatment at 10 days, 30 days, 40 days, and 50 days. For the abrupt treatment, we added 0.5 mL mixture of working solution and 0.5 mL diluted water (overall 1 mL) at 20 days. We added the same volume of acetone, pesticides, and water into each experimental unit. Soil moisture was also monitored every 2 days to ensure the soil water-holding capacity was maintained at 60% ± 5%. Each experimental unit was opened every 10 days for soil moisture adjustment. Microcosms were placed in a dark climate chamber where relative humidity was maintained at 60% and the temperature was kept at 20 °C.

### Harvest and post-harvest measurements

Tea bags were removed from soil microcosms and oven dried. The remaining weight was divided by the initial weight to calculate the rate of decomposition (Keuskamp et al. 2013).

Air-dried soil samples were assessed for aggregate stability (Kemper and Rosenau 1986). First, samples were sieved through a 4-mm sieve, and then 4.0 g of soil was rewetted by capillary action for 5 min. Soils were then placed in a wet-sieving machine (Eijkelkamp, Netherlands) for 3 min (stroke = 1.3 cm, 34 times min^−1^). The remaining fraction was then dried at 60 °C and weighed. Coarse matter consisting of organic debris and sand larger than 0.25 mm was quantified, and the percent water-stable aggregates (WSA) was then calculated via the following formula: % WSA = (water stable aggregates − coarse matter)/(4.0 g − coarse matter).

To quantify the size distribution of soil aggregates, we followed Kemper and Rosenau (1986). Samples were dry sieved through a stack of four sieves, 2 mm, 1 mm, 0.25 mm, and 0.1 mm. The sieves were then moved forward and backward a total of ten times to ensure particle fractionation with minimal abrasion. The mean weight diameter (MWD) in mm was then calculated via the following equation—$MWD= {\sum }_{i=1}^{n}{\overline{x} }_{1{ w}_{i }}$ where ${\overline{x} }_{1}$ is the mean diameter of fraction size i and ${w}_{i}$ is the proportion of total soil mass in fraction size i. Based on this formula, soil aggregates are classed by mean diameter; thus, with increasing MWD, the proportion of large aggregates in the given sample increases.

Soil pH was assessed by mixing 5.0 g of air-dried soil into a 50-mL falcon tube with 10 mM CaCl_2_ at a ratio of 1:5. After mixing with a shaker for ten minutes, samples were centrifuged, and three sub-samples were pipetted from the solution for a triplicate measure per soil sample. The average value was then used for further data analysis.

Soil EC was quantified via a 1:5 soil to water suspension that was placed in a 50-mL falcon tube and shaken for 10 min. Samples were then centrifuged at 3000 rpm at room temperature prior to measurement with a conductivity meter.

### Statistics

Statistics were conducted in R version 4.1.1 (R Development Core Team 2023). To assess the effects of treatments on our response variables, we used the R package “dabestr” (Ho et al. 2019), implementing a bootstrapping method (5000 iterations) to generate unpaired mean differences. Additionally, we used multiple linear regression to analyze relationships between variables and treatments.

Null models were used to predict joint impacts of pesticides on soil processes. Null models were generated in R (version 4.1.1) via modifications of code from Rillig et al. (2019). We tested an additive model, a multiplicative model, which assumes the combined effects can be calculated via the proportional changes caused by each stressor, and a dominative model, which assumes that the effect of all combined stressors is equal to the “dominant” stressor. R was used to generate and compare the 95% confidence intervals and mean differences between model predictions and actual data, estimated by nonparametric bootstrapping with 1000 iterations following the methodology of a prior study (Rillig et al. 2019). All plots were generated either via dabestr or ggplot2 (Wickham 2009).

## Results

### Effects of single and combined application of pesticides

**Fig. 1:**
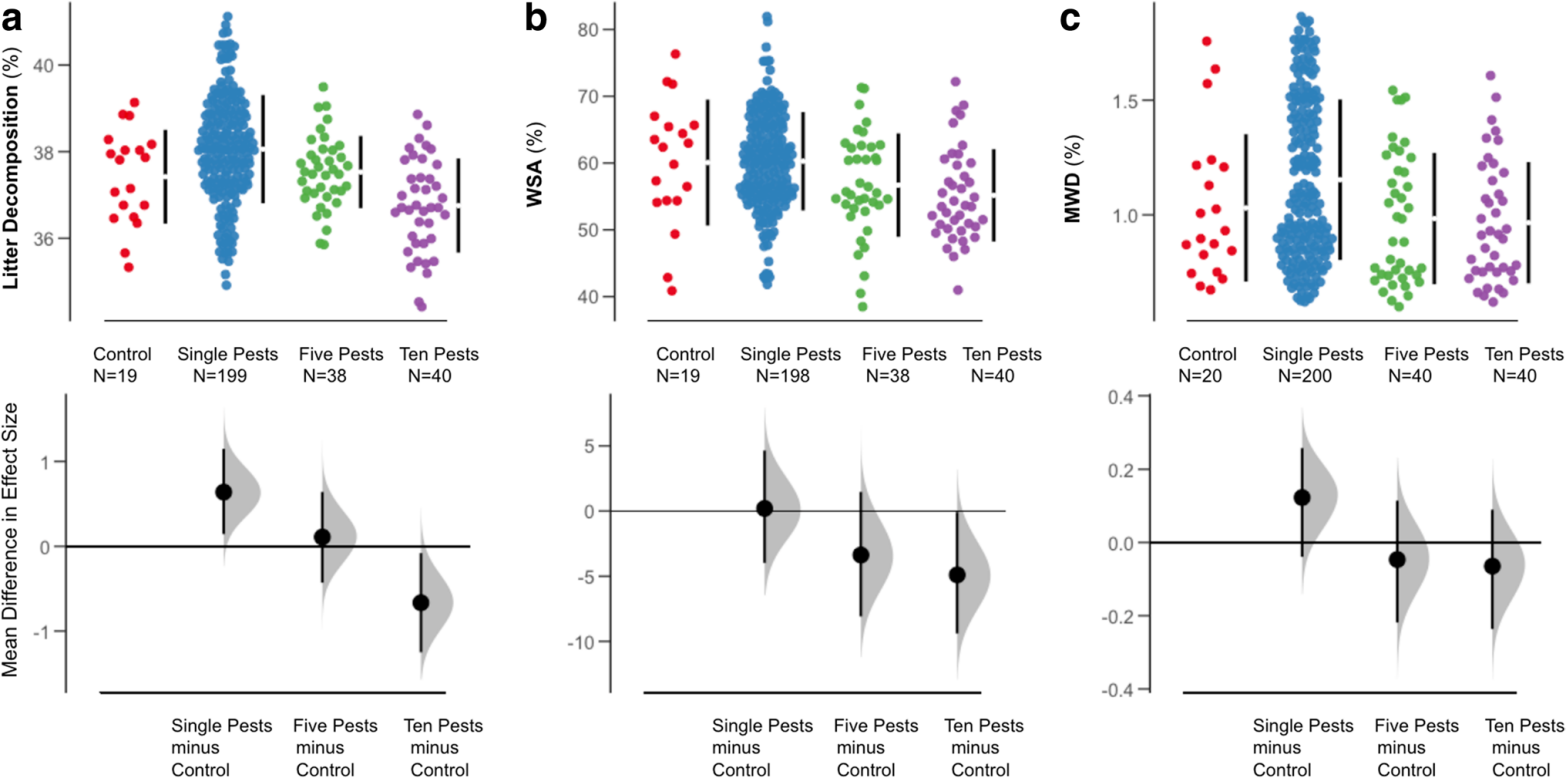
Impact of overall pesticide treatments on soil processes: **a** litter decomposition (LD, %), **b** water stable aggregates (WSA, %), **c** aggregate mean weight diameter (MWD, mm). Top panels are scatterplots of raw data per treatment; the bottom panel shows mean difference in effect sizes compared to the control, specifically: circles represent the bootstrapped effect size mean (effect magnitude) and vertical lines the corresponding 95% confidence interval (effect precision). The density plots depict bootstrapped data distribution

We found negative impacts of pesticide richness when comparing treatments of single (combined data from the single applications), five, and ten combined pesticide treatments. Remarkably, we found that the combined application often had negative effects, while the single applications had positive or neutral effects. Six individual pesticides (diflufenican, napropamide, cyproconazole, s-metolachlor, metrafenone, and clothianidin) all positively affected decomposition when compared to the control with the remaining four exhibiting neutral effects (Figure S2). Furthermore, the five-pesticide combination treatment had a neutral effect when compared to the control, but the ten pesticide combination treatment negatively affected decomposition when compared to the control (Fig. 1). One pesticide, imidacloprid, positively affected WSA in comparison to the control, with the other nine pesticides exhibiting neutral impacts (Figure S2). However, both five and ten pesticide treatments negatively affected WSA when compared to the control (Fig. 1). Two pesticides, napropamide and metrafenone, positively affected MWD in soil samples, with both the five and ten pesticide treatments having neutral effects on MWD when compared to the control (Figure S2, Fig. 1). Increased numbers of pesticides significantly increased soil pH, with the opposite trend revealed for soil EC (Figure S5 and S6). Despite multiple pesticide treatments increasing soil pH, pH was not found to be a significant driver of any of the measured soil processes (Figure S7).

**Fig. 2:**
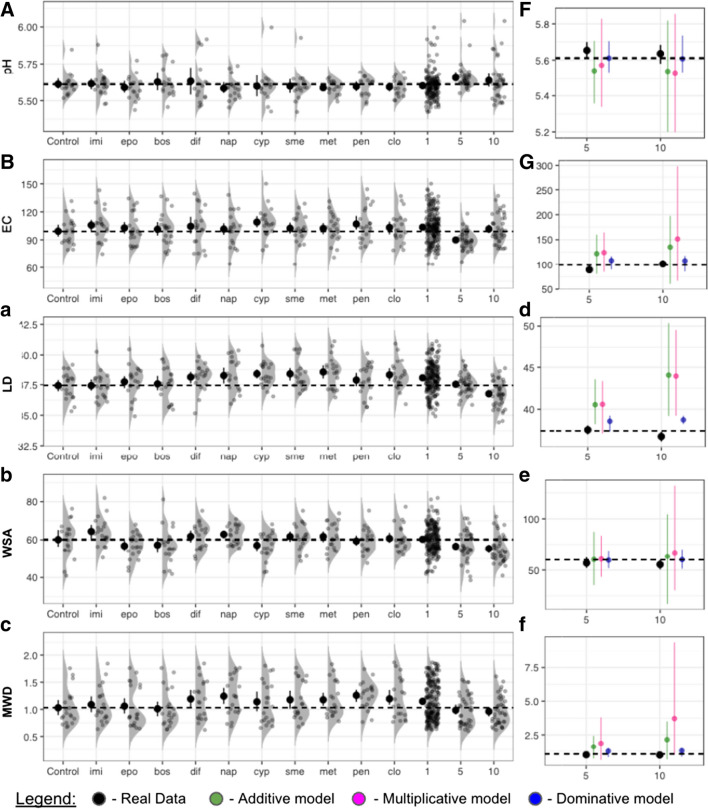
Null modeling of pesticide interactions based on single factor data. Single pesticide effects (**a**–**c**) were used to predict combined effects of pesticide application in the five and ten pesticide application treatments (**d**–**f**). Observed data represented by black circles with a density distribution of the raw data in gray behind each treatment. Error bars of multiple factor interactions were generated by bootstrapped values with 1000 repetitions. Observed data deviated from all major model predictions pointing towards a stronger combined effect of multiple pesticide treatments than predicted by the various null models. Abbreviations as in Fig. 1

To predict the joint effects of pesticides from the single effects, we used three null models to investigate the overall dataset, which includes both abrupt and gradual data (Fig. 2). None of the tested models well-fit the measured data, pointing towards a synergism of pesticides when acting in combination resulting in different effects than those predicted by the null models (Schäfer and Piggott 2018).

### Rate of change

**Fig. 3:**
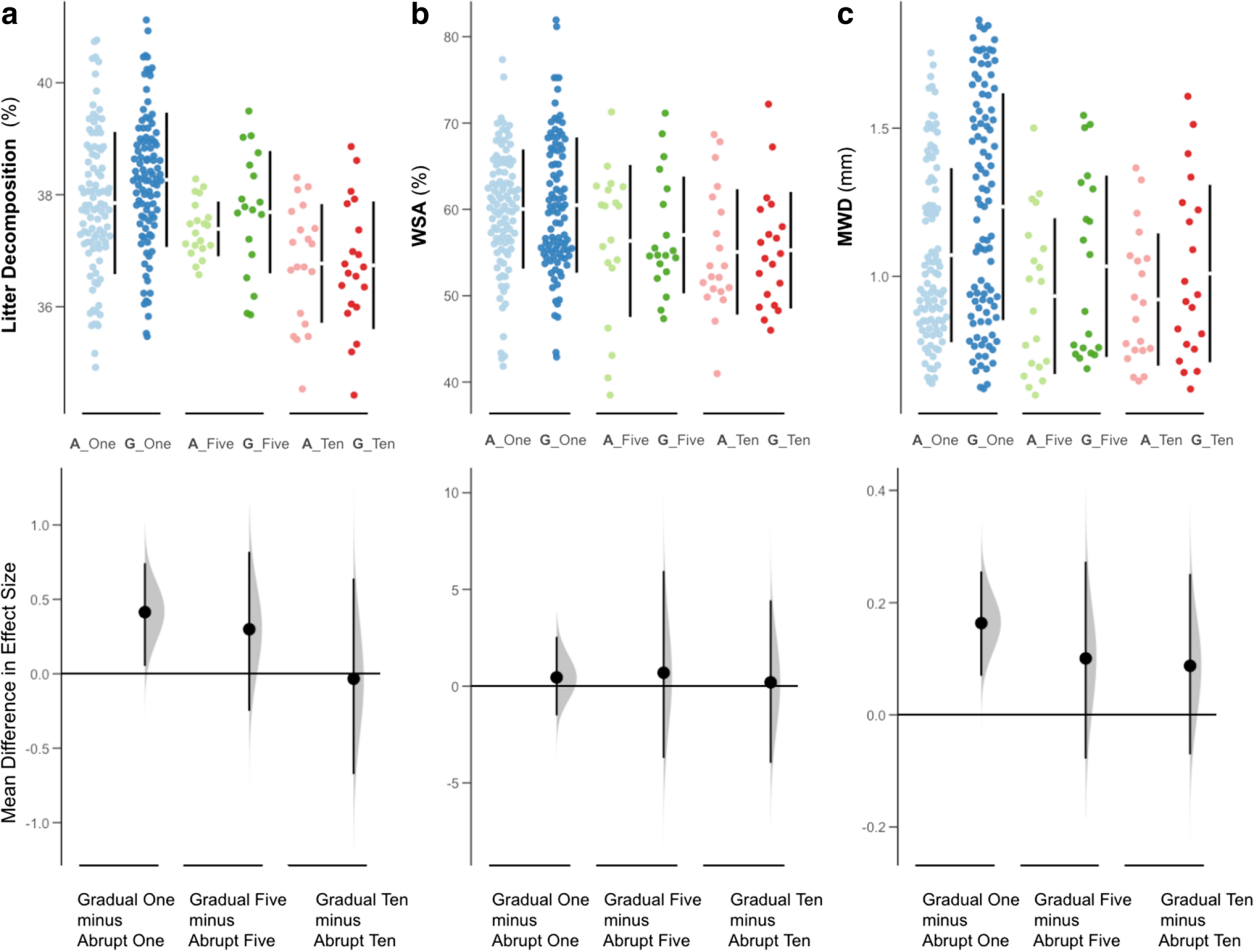
Paired comparisons of gradual vs. abrupt pesticide treatments including single pesticides (A_One / G_One), five pesticides (A_Five / G_Five), and ten pesticides (A_Ten / G_Ten) impact on: **a** litter decomposition (%), **b** water stable aggregates (%), **c** aggregate mean weight diameter (mm). Top panels are scatterplots of raw data, paired per abrupt and gradual treatments; the bottom panel shows mean difference in effect sizes, specifically, the gradual-abrupt treatments, with circles representing the bootstrapped effect size mean (effect magnitude) and vertical lines the corresponding 95% confidence interval (effect precision). The density plots depict bootstrapped data distribution

Negative consequences of both abrupt and gradual pesticide application were observed for all soil processes (Figure S4). In general, increasing the number of pesticide treatments for both abrupt and gradual rates of application resulted in significantly lower rates of litter decomposition, WSA, and MWD. Subsequent comparison of paired differences of abrupt and gradual rates for each pesticide treatment revealed that gradual application of single pesticides had positive effects on decomposition and MWD respectively (Fig. 3). All other paired relationships had neutral effects when compared to each other (Fig. 3 and Figure S3).

## Discussion

Pesticides, when applied individually, can affect both bacterial and fungal populations causing cascading consequences for microbial biomass, enzyme activity, and nutrient cycling (El-Nahhal and El-Hams 2017; Gonzalez-Lopez et al. 1993; Khan et al. 2006; Swissa et al. 2014). Here, we provide evidence for negative impacts of the combined occurrence of pesticides on soil processes including litter decomposition, WSA, and MWD, compared to the effect of pesticides acting individually.

### Single pesticide effects

Decomposition represents a major ecosystem process important for nutrient turnover. In general, all pesticides when applied singly resulted in an increase in decomposition rate. This phenomenon has been previously documented and is likely because many microbial species can utilize certain components of pesticides, including nitrogen, phosphorus, and carbon contained in these molecules, for their own growth and subsequent activity (Sviridov et al. 2015). The microbial ability to utilize these products have also been shown to fluctuate based on the various stages of decomposition of pesticide-based products, meaning different strains of microbes may benefit from these products at various times (Das and Varma 2010). Although pesticides represent a vast array of chemicals, pesticides including fungicides, herbicides, and insecticides in similar chemical classes to those used in this study have been shown to stimulate microbial growth and activity. For example, the neonicotinoid carbofuran increased microbial biomass (Kim et al. 2004), the fungicide carboxamide stimulated the population of *Azospirillum* and other anaerobic nitrogen fixers in soil (Myresiotis et al. 2012), the herbicide aryloxyphenoxypropionate fenoxaprop increased microbial biomass, carbon and nitrogen (Nie et al. 2011), the herbicide chloroacetamide butachlor stimulated anaerobic fermentative and sulfate reducing bacteria (Li et al. 2013), and the herbicides mesotrione and isoproturon increased soil microbial and bacterial counts, respectively (Romdhane et al. 2016; Wang et al. 2010). Pesticides utilized in this experiment have been previously studied and corroborate patterns we find including the stimulated growth of certain bacterial species and rates of litter decomposition in the presence of singly applied pesticides (Cycoń et al. 2013; Książek-Trela et al. 2022; Long et al. 2014; Mohamed 2013, Figure S2.)

Like trends observed regarding microbial decomposition, both proportions of water stable aggregates and MWD increased with the application of single pesticides. Prior studies have shown that fungi play key roles in the formation of macroaggregates (250–2000 µm), while bacteria tend to contribute more to the formation of microaggregates (53–250 µm) (Lynch and Bragg 1985; Lehmann et al. 2017). The contributions of these two microbial groups are thus expected to slow down aggregate turnover and further the formation of new aggregates (Rillig et al. 2015; Six et al. 2004). The addition of single pesticides could have provided the microbial groups key for aggregate formation with substrates they can invest in growth that furthers aggregate formation.

### Multiple pesticide effects

Null models represent valuable tools that can be applied to predict and understand the joint effects of multiple stressors (Schäfer and Piggott 2018). Our testing of three null models revealed evidence of synergism between pesticides, caused by stronger combined effects than those predicted by the various null models. In the five and ten pesticide treatments, we observed a sharp drop-in decomposition rate, compared to the single pesticide treatments. This is due to the synergistic effect of multiple pesticides acting simultaneously, likely by affecting microbial functioning enough to hamper decomposition. Interestingly, one study found that the interplay between two herbicides, metolachlor, and chlorothalonil when applied together led to the increased persistence of metolachlor as opposed to when it was applied individually, which is relevant when interpreting the potential impacts of interacting pesticides in the five and ten pesticide treatments (White et al. 2010). No studies to our knowledge have experimentally investigated the effects of multiple pesticides on soil processes; however, there is literature supporting the trend of multiple factors having a combined negative effect on soil processes and microbial community composition as compared to exposure to only single factors (Reich et al. 2020; Séneca et al. 2020; Rillig et al. 2019; Yang et al. 2022).

In addition to impacting rates of decomposition, multiple pesticides also affected soil aggregation. Soil aggregation represents a fundamental ecosystem process leading to the formation of soil structure. This process is important for water filtration, aeration, soil fertility, carbon storage, and resistance to erosion (Bryan 1968; Tisdall and Malcolm 1982; Boix-Fayos et al. 2001; Li et al. 2021; Kemper and Rosenau 1986). In contrast to the addition of single pesticides, WSA and MWD both decreased in the five and ten pesticide treatments. Again, this is likely due to effects on microbial activity or diversity. In addition to pesticides, both changes in soil pH and EC were assessed in each treatment to evaluate their potential roles in shaping shifts in litter decomposition, WSA, and MWD. Most pesticides added were slightly acidic, and in single pesticide treatments, pH was not markedly increased; however, in the five pesticide treatments, pH levels were more basic as compared to the control, which runs counter to expected trends (Figure S5 and S6). Soil EC was found to be reduced in multiple pesticide treatments, which runs contrary to some results showing pesticide use increases soil EC (Yargholi and Azarneshan 2014). In general, increased rates of soil EC have been found to reduce soil stability and aggregates, and impact microbial enzyme production and decomposition (Singh 2016); thus, changes in EC could have contributed to the observed effects.

### Rate of change

In general, gradual application of pesticides had less of an impact on decomposition rates, WSA, and MWD as opposed to abrupt application. Although there are no studies examining differences between abrupt or gradual pesticide application to our knowledge, other studies have provided evidence that abrupt application of treatments as opposed to more gradual ones can result in more negative effects in fungi and soil (Li et al. 2021). Our results show that gradual application should be included in future protocols examining pesticide effects, since off-target effects of these chemicals in non-agricultural systems are very likely to unfold in such a gradual fashion, which can attenuate effects.

## Conclusion

This research highlights the importance of continued investigation of pesticide effects in soil, especially with their increased rates of use globally. Further research should certainly include investigations into the effects of multiple pesticides, especially, since when only tested alone, many pesticides had minor or even positive impacts, a trend that significantly shifts with combined applications. Ideally, policies both in the EU and the world can target reduction of pesticides by both minimizing input and promoting more sustainable agronomic practices that reduce pesticide use. Furthermore, policies should aim to reduce the influx of new pesticides into the market until they have been thoroughly tested, not only as single active substances, but in relevant mixtures. These points should be considered to make effective policy that adequately protects both natural and agricultural systems. In summary, studies such as this provide a stark reminder of the danger of heavy pesticide usage since a continued reliance on such chemicals is likely to result in significant consequences for soil functions, posing major issues for both humanity and nature in the future.

### Supplementary Information

Below is the link to the electronic supplementary material.

Supplementary file1 (DOCX 831 KB)

## Data Availability

All data utilized in this study can be accessed from the FigShare repository 10.6084/m9.figshare.24961392.
