# Supplementary material for: Combined application of up to ten pesticides decreases key soil processes
Source: Environ Sci Pollut Res Int. 2024 Jan 16;31(8):11995–2004. doi: 10.1007/s11356-024-31836-x (PMC11289034; doi:10.1007/s11356-024-31836-x)
Supplement: Supplementary file 1 — Supplementary file1 (DOCX 831 KB) [file 11356_2024_31836_MOESM1_ESM.docx]

*Supplemental Materials*


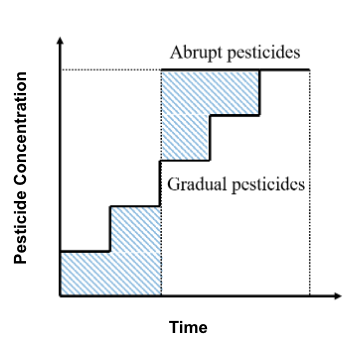


**Supplementary Figure 1** The principle of gradual and abrupt pesticide addition: Maintaining the area of the curve is necessary for both treatments. Gradual application is applied from the beginning of the experiment and added biweekly in increments, opposed to the Abrupt treatment which is applied in a single, high volume dose

**
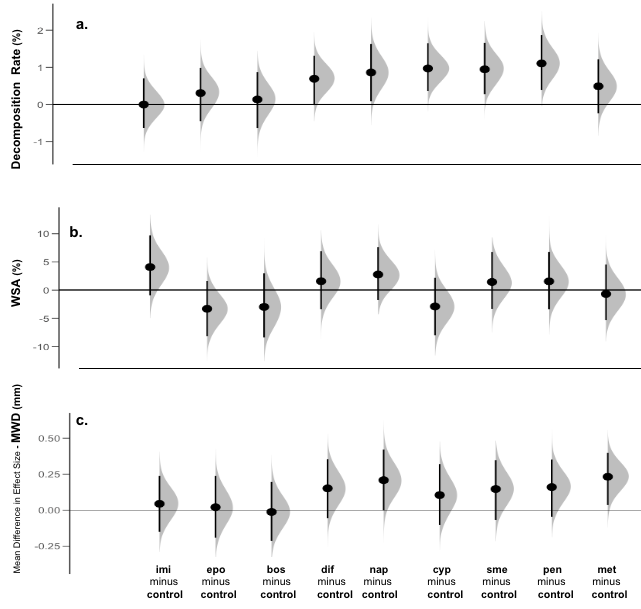
**

**Supplementary Figure 2** Impact of individual pesticide treatments on soil processes: (A). Litter Decomposition (%), (B). Water Stable Aggregates (%), (C). Aggregate Mean Weight Diameter (mm). The panels show a mean difference in effect sizes compared to the control, specifically: circles represent the bootstrapped effect size mean (effect magnitude) and vertical lines the corresponding 95% confidence interval (effect precision). The density plots depict bootstrapped data distribution

**
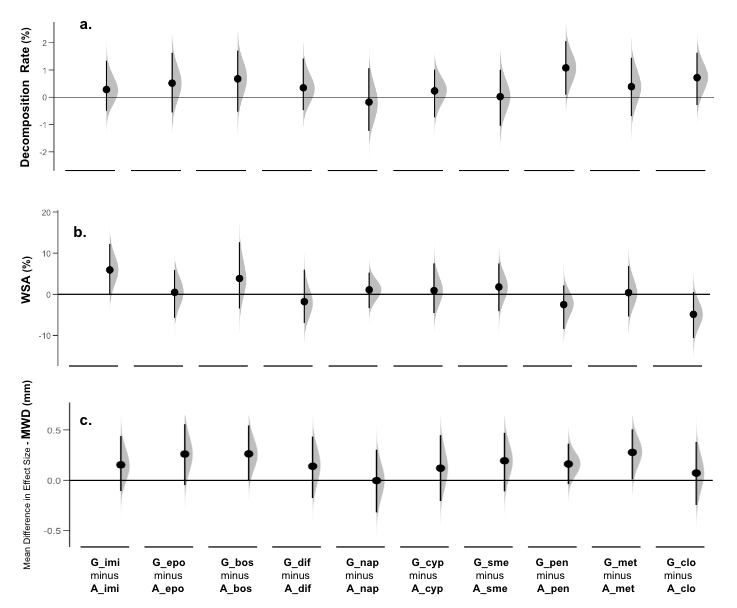
**

**Supplementary Figure 3** Paired comparisons of gradual vs. abrupt individual pesticide treatments on: (A). Litter Decomposition (%), (B). Water Stable Aggregates (%), (C). Aggregate Mean Weight Diameter (mm). The panel shows mean difference in effect sizes, specifically, the gradual - abrupt treatments, with circles representing the bootstrapped effect size mean (effect magnitude) and vertical lines the corresponding 95% confidence interval (effect precision). The density plots depict bootstrapped data distribution


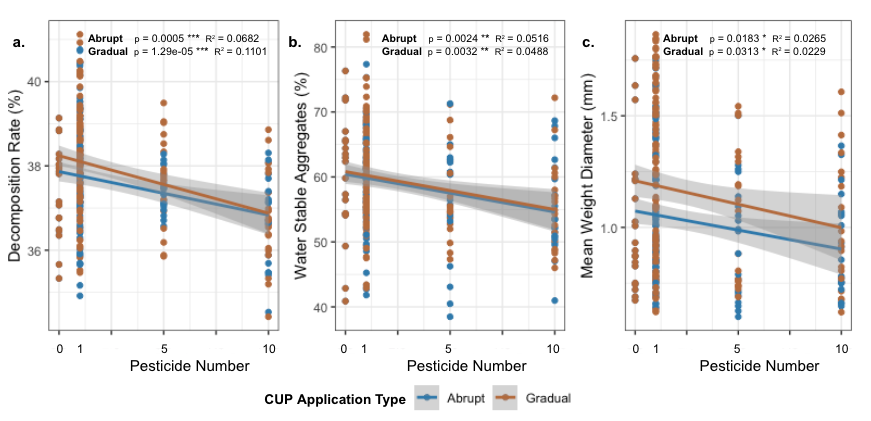


**Supplementary Figure 4** Effects of increasing number of pesticides (control with zero pesticides, one, five and ten pesticides) on the soil process (a) organic matter loss (in %), (b) water-stable aggregates (in %) and (c) mean weight diameter (in mm) under the influence of gradual (brown dots) or abrupt (blue dots) application. Relationships between variables are exhibited via a linear regression fitted between pesticide number and decomposition, WSA, and MWD, with both abrupt and gradual relationships plotted per variable. Significance of the relationship and amount of variation explained are shown by the p and R^2^ respectively


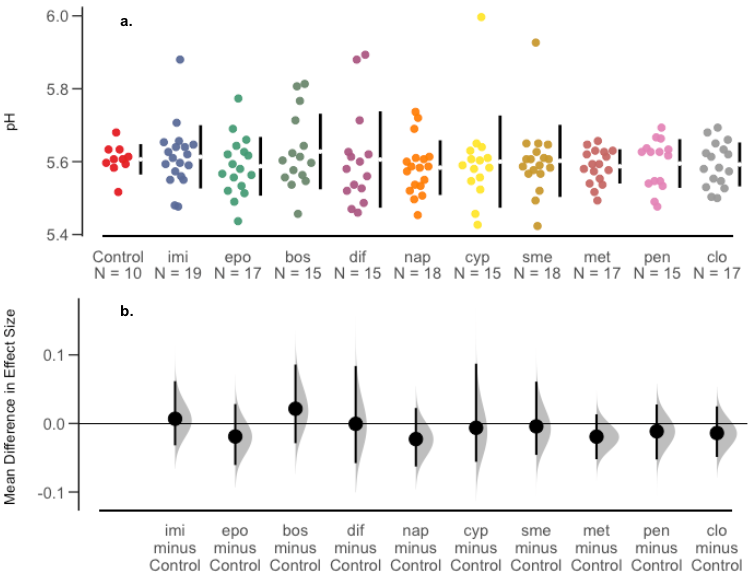


**Supplementary Figure 5** Individual Pesticides and Impact on Soil pH. Top panels are scatterplots of raw data per treatment, the bottom panel shows mean difference in effect sizes compared to the control, specifically: circles represent the bootstrapped effect size mean (effect magnitude) and vertical lines the corresponding 95% confidence interval (effect precision). The density plots depict bootstrapped data distribution

**
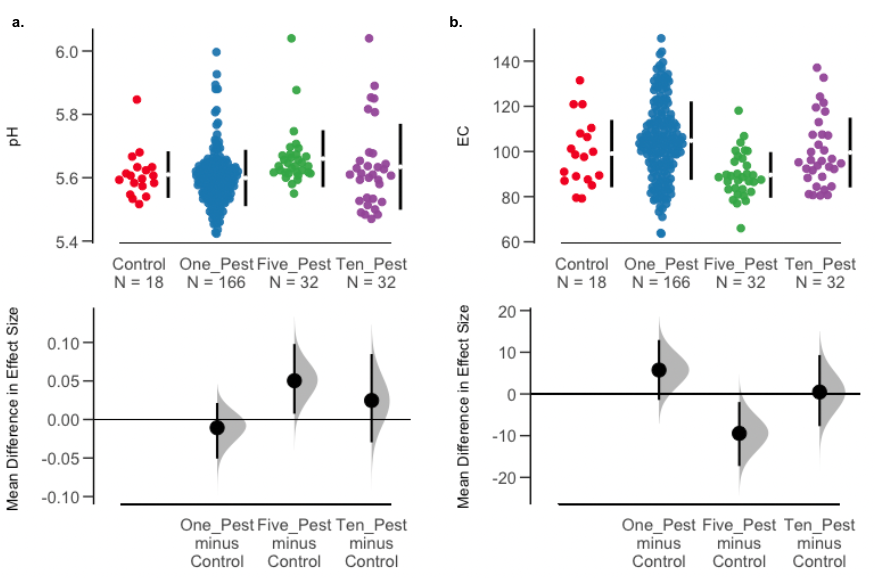
**

**Supplementary Figure 6** Impact of multiple pesticides on soil EC and pH: Impact of pesticide treatments on soil processes: (A). pH (%), (B). EC. Top panels are scatterplots of raw data per treatment, the bottom panel shows mean difference in effect sizes compared to the control, specifically: circles represent the bootstrapped effect size mean (effect magnitude) and vertical lines the corresponding 95% confidence interval (effect precision). The density plots depict bootstrapped data distribution


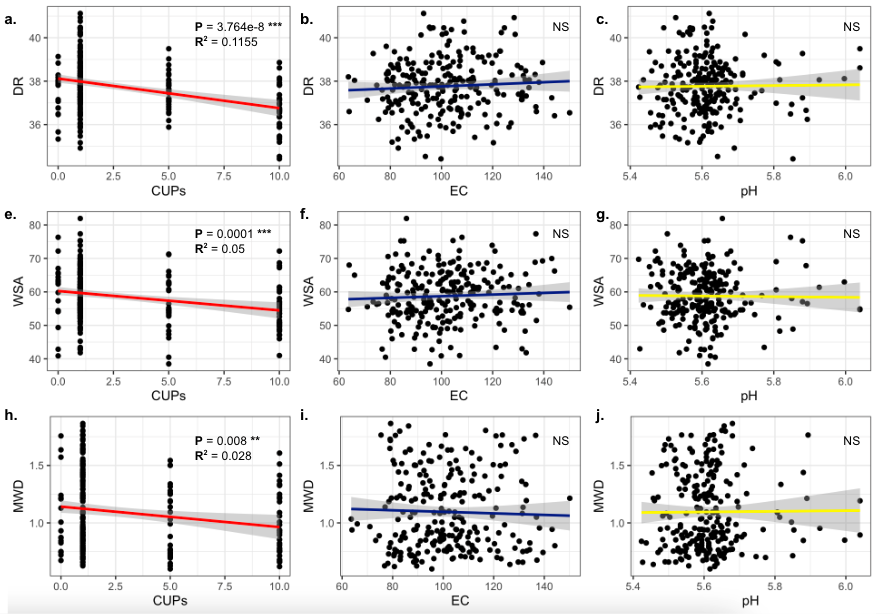


**Supplementary Figure 7**  Impact of pesticides, EC, and pH on Soil Processes: (A). Significant impact of pesticides (viewed on a continuous scale) on decomposition rate. (B). Non-significant (NS) relationship between decomposition rate and soil EC. (C). Non-significant (NS) relationship between decomposition rate and soil pH (D). (E). Significant impact of pesticides (viewed on a continuous scale) on water stable aggregates. (F). Non-significant (NS) relationship between water stable aggregates and soil EC. (G). Non-significant (NS) relationship between water stable aggregates and soil pH (H). Significant impact of pesticides (viewed on a continuous scale) on mean weight diameter. (I). Non-significant (NS) relationship between aggregate mean weight diameter and soil EC. (J). Non-significant (NS) relationship between aggregate mean weight diameter and soil pH

| Rank | Name | pH | Function of pesticides |
| --- | --- | --- | --- |
| 1 | Imidacloprid | Acidic | It acts as a nicotinic acetylcholine receptor agonist, blocking the central nervous system. It disturbs the pest’s motor nervous system and causes the failure of chemical signal transmission, paralysis and death of pests ^[4]^. |
| 2 | Epoxiconazole | Acidic | It acts as a C-14 demethylase inhibitor in sterol biosynthesis, blocking the formation of the cell wall of the bacteria ^[5]^. |
| 3 | Boscalid | Acidic | It acts as succinate coenzyme Q reductase inhibitor in the mitochondrial respiratory chain and has a strong inhibitory ability on spore germination ^[6]^. |
| 4 | Diflufenican | Acidic | It acts as a carotenoid biosynthesis inhibitor, leading to the destruction of chlorophyll, cell rupture, and plant death ^[7]^. |
| 5 | Napropamide | Acidic | It inhibits the synthesis of certain enzymes in the body, so that roots and shoots cannot grow and die (uncertainty of its specific function). |
| 6 | Cyproconazole | Acidic | It acts as a sterol demethylation inhibitor. |
| 7 | S-metolachlor | Neutral | It acts as a cell division inhibitor and mainly inhibits cell growth by inhibiting the synthesis of long-chain fatty acids ^[8]^. |
| 8 | Metrafenone | Acidic | It interferes with development and formation of appressorium during germination of pathogenic bacteria and establishment or formation of polar actin tissue, thereby hindering the normal development and growth of the mycelium. |
| 9 | Pendimethalin | Neutral | It acts as inhibitor of meristem cell division, suppressing the sprouts and secondary roots of weeds ^[9]^. |
| 10 | Clothianidin | Acidic | It works as combining with nicotinic acetylcholine receptors (nAChRs) of the insect’s central nervous system. Immune disrupters can influence in multiple ways the intricate network of interactions among stress agents that have a synergistic impact on health of insects ^[10]^. |

**Table S2**: Functions and characteristics (pH) of pesticides and related references. All pesticides utilized in our study are either acidic or neutral, thus cannot cause acid-base neutralization in solution which will prevent potential inactivation.

| Rank | Name | Type | Family | Solubility- in water at 20 ℃ （mg L^-1^） |  | |
| --- | --- | --- | --- | --- | --- | --- |
| 1 | Imidacloprid | Insecticide | Neonicotinoid | 610 (high) |  |  |
|  |  |  |  |  |  |  |
|  |  |  |  |  |  |  |
|  |  |  |  |  |  |  |
| 2 | Epoxiconazole | Fungicide | Triazole | 7.1 (low) |  |  |
|  |  |  |  |  |  |  |
|  |  |  |  |  |  |  |
|  |  |  |  |  |  |  |
| 3 | Boscalid | Fungicide | Carboxamide | 4.6 (low) |  |  |
|  |  |  |  |  |  |  |
|  |  |  |  |  |  |  |
|  |  |  |  |  |  |  |
| 4 | Diflufenican | Herbicide | Carboxamide | 0.05 (low) |  |  |
|  |  |  |  |  |  |  |
|  |  |  |  |  |  |  |
|  |  |  |  |  |  |  |
| 5 | Napropamide | Herbicide | Alkanamide | 74 (moderate) |  |  |
|  |  |  |  |  |  |  |
|  |  |  |  |  |  |  |
|  |  |  |  |  |  |  |
| 6 | Cyproconazole | Fungicide | Triazole | 93 (moderate) |  |  |
|  |  |  |  |  |  |  |
|  |  |  |  |  |  |  |
|  |  |  |  |  |  |  |
| 7 | S-metolachlor | Herbicide | Chloroaceta-mide | 480 (moderate) |  |  |
|  |  |  |  |  |  |  |
|  |  |  |  |  |  |  |
|  |  |  |  |  |  |  |
| 8 | Metrafenone | Fungicide | Benzopheno-ne | 0.492 (low) |  |  |
|  |  |  |  |  |  |  |
|  |  |  |  |  |  |  |
|  |  |  |  |  |  |  |
| 9 | Pendimethalin | Herbicide | Dinitroaniline | 0.33 (low) |  |  |
|  |  |  |  |  |  |  |
|  |  |  |  |  |  |  |
|  |  |  |  |  |  |  |
| 10 | Clothianidin | Insecticide | Neonicotinoid | 340 (moderate) |  |  |
|  |  |  |  |  |  |  |
|  |  |  |  |  |  |  |

**Table S4:** Environmental fate (mostly solubility in water and organic solvents at 20 °C (mg l⁻¹)) of the selected 10 common pesticides, according to PPDB. Imidacloprid (100 μg/mL acetone) and Clothianidin (15.2 mg/mL acetone) both have a lower solubility in acetone, compared with other pesticides under 20 ℃. Therefore, we will prepare the pesticide solution with low doses of stock solution to ensure they are fully mixed and will refrigerate under 4 ℃, thus avoiding crystallization in a lower environment (-20 ℃).

|  | R1 (2.4.5.9.10) | R2  (3.6.7.8.10) | R3  (1.2.4.6.9) | R4  (1.2.3.6.9) | R5  (1.3.6.9.10) | R6  (1.6.7.8.9) | R7  (2.4.5.8.9) | R8  (1.4.5.6.7) |
| --- | --- | --- | --- | --- | --- | --- | --- | --- |
| 1 | Pendimethalin | Metrafenone | Diflufenican | Pendimethalin | Cyproconazole | Pendimethalin | Diflufenican | S-metolachlor |
| 2 | Clothianidin | Cyproconazole | Epoxiconazole | Boscalid | Imidacloprid | Cyproconazole | Metrafenone | Napropamide |
| 3 | Diflufenican | S-metolachlor | Pendimethalin | Epoxiconazole | Pendimethalin | S-metolachlor | Napropamide | Diflufenican |
| 4 | Epoxiconazole | Boscalid | Cyproconazole | Cyproconazole | Boscalid | Imidacloprid | Epoxiconazole | Cyproconazole |
| 5 | Napropamide | Clothianidin | Imidacloprid | Imidacloprid | Clothianidin | Metrafenone | Pendimethalin | Imidacloprid |

R9: (2.3.5.7.8) 1. Epoxiconazole; 2. Napropamide; 3. S-metolachlor; 4. Boscalid; Metrafenone.

R10: (3.5.6.9.10) 1. Bocalid; 2. Napropamide; 3. Cyproconazole; 4. Clothianidin; 5. Pendimethalin

**Table S5**: different combinations among 10 replicates of all 5 gradual and abrupt
